# Human Ascariasis: Diagnostics Update

**DOI:** 10.1007/s40475-015-0064-9

**Published:** 2015-10-03

**Authors:** Poppy H. L. Lamberton, Peter M. Jourdan

**Affiliations:** Imperial College London, St Mary’s Campus, Norfolk Place, London, W2 1PG UK; Schistosomiasis Control Initiative, Imperial College London, St Mary’s Campus, Norfolk Place, London, W2 1PG UK

**Keywords:** Ascariasis, *Ascaris*, Diagnosis, Microscopy, Immunology, PCR

## Abstract

Soil-transmitted helminths (STHs) infect over one billion people worldwide. Ascariasis may mimic a number of conditions, and individual clinical diagnosis often requires a thorough work-up. Kato-Katz thick smears are the standard detection method for *Ascaris* and, despite low sensitivity, are often used for mapping and monitoring and evaluation of national control programmes. Although increased sampling (number of stools) and diagnostic (number of examinations per stool) efforts can improve sensitivity, Kato-Katz is less sensitive than other microscopy methods such as FLOTAC®. Antibody-based diagnostics may be a sensitive diagnostic tool; however, their usefulness is limited to assessing transmission in areas aiming for elimination. Molecular diagnostics are highly sensitive and specific, but high costs limit their use to individual diagnosis, drug - efficacy studies and identification of *Ascaris suum*. Increased investments in research on *Ascaris* and other STHs are urgently required for the development of diagnostic assays to support efforts to reduce human suffering caused by these infections.

## Introduction

Soil-transmitted helminths (STHs) infect over 1.45 billion people worldwide, with an estimated 819 million individuals infected with *Ascaris lumbricoides*, 465 million with *Trichuris trichiura* and 439 million with hookworm (*Necator americanus* and/or *Ancylostoma duodenale*) [1]. Single- and multi-species infections cause human disease ranging from mild to severe and even fatal cases, as well as increased school absenteeism, although this might not be detectable at a community level [2]. Most of such neglected tropical diseases (NTDs) occur in areas with poor sanitation and hygiene; however, increased travel and migration have made STH infections more common also in non-endemic areas.

The World Health Assembly, together with endemic countries, donors and drug-donating pharmaceutical companies, have set ambitious goals for the control of STH-related morbidity by 2020, aiming to treat at least 75 % of school-age children and high-risk groups, with mass drug administration (MDA) of albendazole or mebendazole [3, 4]. Sensitive, specific, user-friendly and cost-effective diagnostic tests are imperative for individual diagnosis and for planning, monitoring and evaluation (M&E) of mass ‘preventative chemotherapy’ programmes, and novel tools are needed, especially for measuring decreased infection intensities and drug efficacy [3]. With the scale-up of national STH - control programmes, the associated scientific opportunities and known limitations of the currently recommended techniques, research on *Ascaris* diagnostics is needed more than ever.

We review the available literature for the diagnosis of *A. lumbricoides* infection and discuss the research and field trials that inform current and potential future diagnostic assays. We cover scenarios ranging from clinical settings to large-scale control programmes, and emphasise the need for integration of diagnosis of multi-species infections.

## Methods

We searched the databases PubMed, Google Scholar, Web of Science and EMBASE for all publications on diagnostic techniques of *Ascaris* using combinations of *Ascaris*/Ascariasis/*A. lumbricoides*/soil-transmitted helminths/STH/helminth and diagnostics/diagnosis/sensitivity/specificity/Kato-Katz/FLOTAC/ethyl/midi/ether/antigen/immunology/immunoglobulins/LAMP/loop/polymerase chain reaction/PCR/FECPACK/2010/2011/2012/2013/2014/2015, and searched for individual publications by title and/or authors when necessary. Three hundred and sixty-eight papers were retained based on titles, 146 articles were read after screening of abstracts, and the final number of references was limited according to the publisher’s guidelines.

## Clinical Presentation

**Fig. 1:**
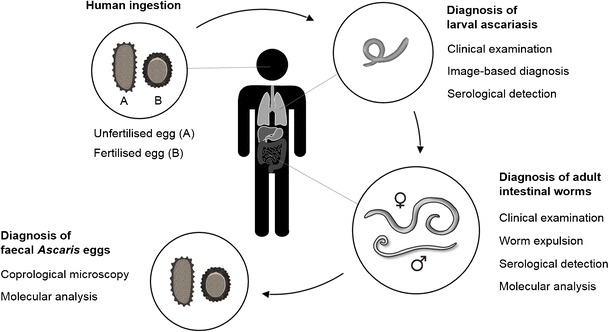
*A. lumbricoides* life cycle and diagnostic markers of infection. After being swallowed, an *A. lumbricoides* larva hatches from the infective egg*, migrates into the vascular system and is transported through the portal veins and right side of the heart to the pulmonary circulation. Unable to cross the capillary network, the parasite penetrates the walls of the alveoli, migrates to the larynx and is swallowed, ending up as an adult worm in the small intestines. The female parasite lays tens of thousands of eggs daily that, through stool excretion, enter the environment and may infect other human hosts. The time from egg ingestion to larval migration takes 10 to 14 days, with egg production starting from 2 to 3 months. Adult worms can live in humans for 1–2 years [5]. *Only fertilised eggs may become infective

*A. lumbricoides* is a parasitic nematode that causes two main forms of pathology: immune-mediated reaction to migrating larvae and nutrient depletion and/or obstruction due to physical presence of adult worms in the gastrointestinal tract [5] (Fig. 1). Infection is often asymptomatic and may occur alongside other diseases. Ascariasis may present as a differential diagnosis to a wide range of conditions (Table 1).

**Table 1:** Differential diagnoses to ascariasis morbidity in humans, grouped by larval and intestinal stages of infection

Findings	Differential diagnoses
Larval ascariasis^a^	
Urticarial, other rash	*Allergy*, *drug reactions*, *infections,* including other parasites, *environmental causes*, *dermatological conditions*
Tender hepatomegaly	*Infections*, including intestinal ascariasis (see below); other parasitic infections, including malaria, amoebiasis, echinococcosis; bacterial infections, including enteropathogenic bacterial abscesses, typhoid and paratyphoid fever; viral infections, including EBV, CMV, HIV and hepatitis; fungal infections *Tumours*, including metastatic or (less common) primary hepatocellular carcinoma; haemangioma; polycystic disease; lymphoma *Vascular causes*, including congestive heart failure; haemolytic disorders; Budd-Chiari syndrome *Toxicity*, including alcoholism and other toxic substances *Metabolic*, including congenital deficiencies such as haemochromatosis, glycogen storage disease; amyloidosis
Cough, dypnoea	*Pulmonary infections* and/or *inflammation*, including infections with other parasites, pneumonia, lung abscess, bronchiectasis, asthma, allergy, COPD, cystic fibrosis, sarcoidosis *Tumours*, including primary or metastatic neoplastic tumours *Vascular causes*, including congestive heart failure, pulmonary embolism, coronary artery syndrome, anaemia *Mechanical causes*, including pneumothorax
Eosinophilia	*Other parasitic infections*, *allergy*, *drug reactions*, rare *congenital* or *malignant diseases*
Increased IgE titres	*Other parasitic infections*, *allergy*, *drug reactions*, rare *congenital* or *malignant diseases*
Intestinal ascariasis^a^	
Acute abdominal pain	*Infection* and/or *inflammation* ^b^, including appendicitis, cholelithiasis/cystitis, pancreatitis, diverticulitis, peritonitis, pyelonephritis *Vascular causes*, including intestinal ischaemia, abdominal aortic aneurysm, sickle cell disease crisis *Other*, including acute adrenal insufficiency, ectopic pregnancy, ovarian torsion, endometriosis, physiological
Ileus	*Bowel obstruction* due to American trypanosomiasis (Chagas disease), constipation, adhesions, hernia, volvulus, intussusception, tumours, IBD, congenital disorders *Intestinal paralysis* due to post-surgical paralytic ileus, drugs, acute pancreatitis or systemic disease
Acute pancreatitis	*Acute pancreatitis*, other causes of acute abdominal pain (see above)
Acute cholecystitis	*Acute cholecystitis*,other causes of acute abdominal pain (see above)
Liver abscess, cholangitis	*Infections*, including other parasites, enteropathogenic bacteria and opportunistic infections associated with AIDS; *cholelithiasis*; *tumours*

*EBV* Epstein-Barr virus, *CMV* cytomegalovirus, *HIV* human immunodeficiency virus, *COPD* chronic obstructive pulmonary disease, *IBD* inflammatory bowel disease, *AIDS *acquired immune deficiency syndrome

^a^In individuals with a high exposure to infection, elements from both stages may co-exist

^b^Apart from appendicitis, conditions most commonly affect adults

Similar to a number of parasite infections, individual diagnosis of ascariasis often depends on a thorough investigation that may include travel history or origin from endemic countries (when presenting in non-endemic areas) and clinical and laboratory examinations, including potentially serological, molecular and image-based diagnostics. Recent findings suggest that ascariasis should be suspected in patients with relevant symptoms even without travel to *A. lumbricoides* endemic areas, as *Ascaris suum*, a species that commonly infects pigs, may also infect and cause pathology in humans [6].

### Migrating *Ascaris* Larvae.

Löffler syndrome, or eosinophilic pneumonitis, is an immune-mediated type I hypersensitivity reaction to larvae migrating through the pulmonary tissue and typically occurs in initial or intermittent infections [7]. Following an incubation period of 4 to 16 days, patients present with fever, cough and dyspnoea. Clinical findings may include urticaria or other rash, abnormal breath sounds by auscultation and tender hepatomegaly. The leukocyte differential count typically reveals eosinophilia, and the chest X-ray may show pulmonary infiltrates. Serology can aid the diagnosis, especially if egg excretion has not yet started, although cross-reactivity with other parasites is common. The syndrome may last up to 3 weeks and can ultimately be fatal. Rarely, *Ascaris* larvae migrate to ectopic sites, and associated eosinophilia may cause complications [8].

### Adult *Ascaris* Worms

Light infections are frequently asymptomatic, whereas heavy infections commonly lead to acute abdominal pain and ileus from conditions such as mechanical small bowel obstruction, volvulus and intussusception, especially in children [5, 9]. In endemic countries, intestinal ascariasis is also a common cause of hepatic, biliary and pancreatic disease, including acute pancreatitis and cholecystitis [10]. Ultrasonography, abdominal X-ray, computed tomography and magnetic resonance imaging scans may identify the cause [11–13]. Endoscopic retrograde cholangiopancreatography may be both diagnostic and therapeutic, and capsule endoscopy can be considered, even in individuals with negative conventional gastrointestinal endoscopy [14, 15].

In endemic countries, *Ascaris* infection is a common cause of malabsorption, and undernutrition and micronutrient deficiencies may lead to growth failure and cognitive impairment, as well as defective immune regulation and increased risk of other parasitic infections [16, 17].

## Coprological Diagnosis

Quantifying the worm burden of *A. lumbricoides* in stool following treatment is time-consuming and cumbersome, and detection of eggs by light microscopy remains the mainstay for diagnosis. The various microscopy-based techniques are also commonly used for other intestinal parasites.

Kato-Katz thick smear [18] is currently the recommended method by the World Health Organization (WHO) for detection of STH infections in endemic areas [3]. For intensity of infection, measured as number of eggs per gram of stool (EPG) [3], Kato-Katz correlates well with worm burden [19]. Kato-Katz slides are relatively cheap and simple to prepare, produce few false positives and allow detection of several co-endemic intestinal parasite species [20•]. However, the high variance of EPG from repeated Kato-Katz sampling, with non-random egg distribution (within the same stool) and daily fluctuations in egg detection (from different stools from the same person, and potentially from mislabelled stools from different people), is an important limitation of this technique [19]. Due to the high variance, probably exacerbated by the small fixed volume of stool used (normally 41.7 mg), Kato-Katz has limited sensitivity at lower intensities of infection [21, 22•]. In addition, the number of eggs recorded in each smear is multiplied to calculate EPG, but the volume-to-weight ratio is affected by stool density, and actual weights vary considerably [23]. Finally, the diagnostic accuracy of Kato-Katz depends on sufficiently well-trained laboratory technicians.

Increased sampling from one to multiple slides from stools collected on consecutive days greatly improves the sensitivity of Kato-Katz, often resulting in higher prevalence estimates [24–26]. Three days of two Kato-Katz slides per stool is sufficient to reach ≤1 % false negative diagnostics for *A. lumbricoides* in a moderate prevalence setting, in comparison with up to 20 smears required for *T. trichiura* [24]. On the other hand, multiple smears do not always improve sensitivity, may bias results through age-related non-compliance [27] and require increased human and financial resources.

FLOTAC® is more sensitive than a single [21, 28] or multiple [22•, 29, 30] Kato-Katz slide/s, possibly due to the larger volume of processed stool (1 g). FLOTAC® could therefore be a useful tool for mapping and monitoring integrated control programmes and for surveillance in low-endemic areas. However, FLOTAC® requires a centrifuge, lacks 100 % sensitivity and often results in reduced egg counts [22•, 28, 29, 31].

The Mini-FLOTAC is a simpler test which does not require expensive equipment or an energy source and has been found to be at least as sensitive as Kato-Katz for determining STH infection intensities across a number of different settings [22•, 32–34]. The choice of flotation solution (FS) for both FLOTAC® and mini-FLOTAC affects species-specific diagnosis, with FS2 recommended for hookworm [32], FS7 for *Schistosoma mansoni* and *A. lumbricoides* [32, 33, 35, 36] and FS4 for all STHs [37].

Mini-FLOTAC has been found to be more expensive [34] yet quicker [33] than Kato-Katz in low-intensity infections following treatment. However, cost per detected case increases as prevalence decreases [34]. Although FLOTAC® is more expensive than Kato-Katz [37], a single FLOTAC® is cheaper, and more sensitive, than triplicate Kato-Katz slides [31], and may be the most suitable coprological technique for accurate prevalence diagnostics in the field.

The McMaster egg counting technique provides accurate estimates of EPG [21], is very easy to use [23] (1-day training) and provides the most reliable estimates of drug efficacies (see below) [38••]; however, it is not as sensitive as FLOTAC [21].

Other diagnostic techniques have shown promising results for *A. lumbricoides*, including the TF-Test® [39], Baermann-Moraes [39], Paratest [40], formalin-ethyl acetate sedimentation [41], sodium acetate formalin (SAF) [42], Hoffman-Pons-Janer [39, 40] and the spontaneous sedimentation in tube technique (SSTT) [43]. In contrast, other methods have shown less promise, such as the Midi Parasep® [41]. Further studies are needed to determine the diagnostic value of these tests for *Ascaris* and other intestinal parasite infections.

### Diagnosis in Infants.

Kato-Katz has low sensitivity for detection of *A. lumbricoides* in breastfed infants, who have more liquid stools and, if infected, lower EPGs than older children [44]. Modified Wisconsin floatation and simple gravity sedimentation are more sensitive for infants than Kato-Katz, formal-ethyl acetate sedimentation or modified formal-ethyl acetate sedimentation [44]. The gravity sedimentation method is labour-intensive but can distinguish fertilised from unfertilised *Ascaris* ova and is unaffected by diarrhoeal stool, unlike the Wisconsin method [44].

### Drug Efficacy.

Statistical simulations indicate that McMaster and Kato-Katz provide reliable estimates of drug efficacy and are suitable for M&E of control programmes [23]. FLOTAC® has also been shown to be more sensitive than Kato-Katz post-treatment for the detection of all three main STHs, particularly when performed on preserved samples [21]; however, this is in contrast to other studies where helminth egg recovery decreases with preservation time [31]. The sensitivity of Kato-Katz and McMaster decreases following anti-helminthic treatment, whereas FLOTAC® remains high [21].

### Diagnosis Using Mobile Phone Technology.

Mobile devices have been adapted for examination of Kato-Katz slides and can accurately diagnose helminth eggs in moderate- to high-intensity infections, with a sensitivity of 81 % for *A. lumbricoides*, but lower for other STHs [45]. It is probable that mobile, lens-free devices, in combination with digital image analysis, may improve stool-based point-of-care diagnosis, particularly with further technological and software development [46•, 47, 48].

## Serological Diagnosis

Detection of antibodies or antigens could provide a simpler, more rapid diagnosis of *Ascaris* infection than conventional stool microscopy. Point-of-care tests are available for other NTDs such as lymphatic filariasis (LF) [49••] and schistosomiasis [50]; however, currently, no such tests exist for STHs.

### Humoural Immune Response.

*A. lumbricoides* generates an antibody production that varies with exposure and intensity of infection, particularly in high-endemic areas [51, 52]. Importantly, factors such as age, genetic predisposition, atopy, nutritional status and co-infections may affect the humoral response to *Ascaris* [17, 53, 54]. Total immunoglobulin (Ig) titres are associated with worm burden in individuals living in endemic areas [51]. In some studies, certain Ig isotypes, such as IgG4, have been found to be sensitive and specific markers of chronic *A. lumbricoides* infection, and to positively correlate with intensity of infection [55, 56]. These findings are consistent with other parasite infections [57], although others have found more variable results for *Ascaris* [52].

Cross-reactivity of anti-*Ascaris* antibodies with epitopes of other helminths is common [51], and standardisation of *Ascaris* antigens for research and diagnostic purposes is warranted, including recombinant antigens, *Ascaris*-associated allergens and antigens of other ascarid species [56, 58, 59].

### Antibodies as Markers of Active Infection.

Few studies have evaluated the use of serological diagnosis of *Ascaris* at the community level. In one study of individuals with high-intensity infections treated repeatedly over several months, anti-*Ascaris* IgG4 fell to levels equal to negative controls [60]. In another study, however, antibody titres did not correlate with worm - load expulsion after treatment [56].

Anti-*Ascaris* antibody titres have been associated with larval stage ascariasis in particular and may remain elevated for several months, even following treatment, especially in areas where re-infection is frequent [7, 60]. Anti-*Ascaris* antibodies are therefore generally not seen as suitable to detect active *Ascaris* infection and could overestimate the number of individuals in need of treatment in mass control programmes. A number of commercial diagnostic tests are available for detection of anti-*Ascaris lumbricoides* IgG and IgM; however, to our knowledge, most are based on somatic *A. lumbricoides* worm antigens and frequently cross-react with other helminths. Interestingly, saliva-based detection of IgG performed well in high-intensity *T. trichiura* infection, but not in *Ascaris* infection [61].

### Antigen Detection.

Whilst antibody detection could represent past infection or exposure, as well as current infections, antigen detection only represents current infections. We did not find any studies of detection of antigens in blood or other specimens for *A. lumbricoides* infection. Detection of schistosome antigen in urine is highly sensitive and is available as a commercialised point-of-care test [50, 62]. Similar tools for STHs may be limited by the location of the adult worms in the intestines, rather than in the blood vessels as is the case for schistosomes, and it is possible that coproantigen tests would be more sensitive than urine or blood tests for STHs.

### Serological Diagnosis in Children.

As control programmes potentially move towards elimination of STHs, antibodies may provide a good marker of infection in young children, especially in areas where children are frequently exposed to intestinal pathogens [63, 64].

### Biomedical Markers.

Few studies have identified biomedical target markers for *A. lumbricoides* infection. Fatty acid products of *A. lumbricoides* infection may be detected in urine by gas-liquid chromatography, and the levels correlate well with worm burden [65]; however, to our knowledge, no such tests are currently available as commercial products.

## Molecular Diagnosis

Molecular diagnostic tools are highly sensitive and specific, and rapid advances are being made, resulting in reduced costs and improved techniques such as real-time quantitative PCR (qPCR) and multiplex assays.

The DNA extraction and amplification of the nuclear first internal transcribed spacer region (ITS1) from single *Ascaris* eggs have primarily been optimised for population genetic analyses [66]. These techniques, used on stool samples, could enable highly sensitive detection of *Ascaris*, particularly by amplification of DNA from single eggs. Methods to detect small amounts of ancient DNA, such as molecular paleoparasitological hybridization approach [67], may improve sensitivity for very low infections.

Multiplex PCR enables the detection of multiple parasite species in a single reaction and can simplify diagnostics by replacing several individual tests with one molecular test. High-throughput PCR assays have been developed, and a multiplex PCR showed promising results for *A. lumbricoides*, *T. trichiura* and *N. americanus* [68].

Unlike conventional PCR, which can only indicate presence of infection, qPCR enables quantification of amplicon (and associated infection intensity). High intensity reactions result in rapid amplification and early fluorescence. qPCR is more sensitive than Kato-Katz and the flotation technique (FS7) for detection of *A. lumbricoides* infections and co-infections [69, 70••, 71]. Multiplex qPCR assays have successfully detected *A. lumbricoides* infection alongside multiple intestinal parasites [69, 70••, 72], with over 90 % of children under 10 years of age harbouring two or more parasites [72]. These findings highlight the importance of multi-species diagnostic tests, even in young children, including common intestinal infections that are often neglected by control programmes. All primers had high sensitivity and specificity, and the quantified DNA correlated strongly with EPG [70••], indicating its potential for measuring parasite reduction following anti-helminthic treatment [70••].

Alternatively, amplicons for several STHs and protozoa can be hybridised to beads for probe-based detection on a Luminex platform providing a high-throughput diagnostic tool with less equipment required than for qPCR [73]. Further, reverse transcriptase PCR can identify specific stages of schistosomes [74] and could be useful for distinguishing new and treatment-resistant *Ascaris* infections.

The significantly higher sensitivity of qPCR over stool microscopy typical for a number species is not always observed for *Ascaris* due to high egg output and technical challenges related to isolating parasite DNA from the resistant, four-layered *Ascaris* egg shell [75]. This may limit the usefulness of future PCR methods such as USB DNA-chip technology [76] for field diagnosis of ascariasis. Also, specific multiplex assays are limited to the species targeted in the respective tests, and DNA from high-intensity infections will compete for dNTPs, thereby deterring detection of species of lower infection intensities. Finally, the price of molecular diagnostic techniques limits its use in endemic areas [69, 70••], and until equipment costs decrease, other diagnostics may remain more cost-effective.

## Parallels to Diagnostic Tools for *Ascaris suum*

Recent studies suggest that *A. suum* may be a relatively common cause of infection in humans, also in areas non-endemic for *A. lumbricoides*, and tools for species-specific diagnosis are required on a larger scale than previously anticipated [6, 77–82]. The zoonotic potential of *Ascaris* spp. may be reinforced by drug resistance to anti-helminthic treatment in domestic pigs [83] and could change public health strategies [77]. Although adult *A. lumbricoides* and *A. suum* worms differ in structure [84], the absence of differences in egg morphology makes stool-based species diagnosis difficult.

### Serological Diagnosis

An *A. suum* antigen-based immunoblot assay was developed that successfully diagnosed human visceral larva migrans (VLM) syndrome assumed to be caused by *A. suum* [85]. An enzyme-linked immunosorbent assay (ELISA) using the *A. suum* haemoglobin antigen correlates well with EPG and worm load, is more sensitive than microscopy and has low cross-reactivity with *Trichuris suis* in experimentally infected pigs [86]. Although developed as a veterinary tool, it could be useful for rapid, multi-species diagnosis in human *Ascaris* infection [87•].

### Molecular Diagnosis

Although PCR may detect a single *Ascaris* egg, it does not appear to discriminate *A. lumbricoides* from *A. suum* [88]. Additional studies from sympatric populations using multi-locus genotype data are required to determine if cross-transmission is a global issue, and to determine what diagnostics are required. Detailed comparisons [89] of the published mitochondrial genome of *A. lumbricoides* [90] and *A. suum* [91] and the complete *A. suum* [92] and *A. lumbricoides* genome (Wellcome Trust Sanger Institute for the 50 Helminth Genomes Initiative) may reveal genes suitable for differentiating infections. However, the mitochondria vary by only 1.9 % [89] and differentiation may not be possible if they are in fact not two distinct species [93, 94].

## Discussion

Despite global efforts to control STH-related morbidity, only approximately 30 % of children worldwide in need of treatment are currently receiving preventive chemotherapy [4]. Key factors for optimal planning, M&E and surveillance of control programmes include accurate diagnostic tools and optimal survey protocols with appropriate sample sizes, number of repeated measurements and timing. The choice of diagnostic technique and protocol will vary depending on the research question being addressed. To date, the development of diagnostic tests for ascariasis has been limited by largely insufficient investments and is further complicated by the fact that no true gold standard exists for comparison of tests. There is a need for tests which compare adult worm expulsion (for up to a week), repeated Kato-Katz and/or other tests that estimate EPG, and PCR, to standardise analyses of current and future diagnostic methods. Ideal relationships should be linear, with low variance. We have reviewed the published literature to identify currently available diagnostic tests that may support endemic countries to achieve global targets, and below we provide our recommendations for each of the components of such control programmes (summarised in Table 2).

**Table 2:** Characteristics of the most common current and potential laboratory-based diagnostic techniques, and their use in national control programmes

	Strengths and limitations					Recommendations for use in control programmes				Integration with other NTDs	
	Spec	Sens	Field-based	Cost	Sample^a^	Mapping	M&E	Drug efficacy	Surveillance^b^	STHs	Common intestinal pathogens
Coprological											
Kato-Katz	✓✓✓	✓	✓✓	✓✓✓	F	✓✓	✓	✓		✓✓✓	
McMaster	✓✓✓	✓	✓✓✓	✓✓✓	F	✓✓	✓	✓		✓✓✓	
FLOTAC^c^	✓✓✓	✓✓	✓	✓✓	F	✓✓	✓✓	✓		✓✓✓	✓✓
Mini-FLOTAC^c^	✓✓✓	✓	✓✓	✓✓	F	✓				✓✓✓	✓✓
Serological											
Antibodies	✓✓	✓✓	✓	✓	B				✓✓✓	✓	✓✓
Antigens^d^	?	?	✓✓✓	✓✓	?^e^	✓✓	✓✓	✓✓	✓✓	✓✓	✓✓
Molecular											
PCR	✓✓✓	✓✓✓	-	✓	F/B	✓	✓	✓	✓✓✓^f^	✓✓✓	✓✓✓
qPCR	✓✓✓	✓✓✓	-	✓	F/B		✓✓	✓✓✓	✓✓^f^	✓✓✓	✓✓✓

*Spec* specificity, *Sens* sensitivity, *M&E* monitoring and evaluation, *NTDs* neglected tropical diseases, *STHs* soil-transmitted helminths, *qPCR* quantitative PCR

^a^F = faeces, B = blood/serum

^b^Surveillance for elimination and/or recrudescence

^c^Choice of flotation solution affects diagnostic accuracy of different species, with FS2 recommended for hookworms, and FS7 for *A. lumbricoides* and *S. mansoni*. Duplicate FLOTAC® using two different flotation solutions is recommended in areas where multiple species co-exist

^d^No antigen tests are available; however, antigen detection has the potential for accurate, non-invasive and rapid diagnosis of active infection

^e^Future diagnostic tools based on non-invasive specimens such urine or oral fluid may be highly applicable for field use, as well as coproantigen tests

^f^Analysis of pooled samples in order to reduce costs

### Geographical Mapping of Disease Distribution

#### Currently Available Tests

Mapping of disease for defining appropriate frequency of MDA is currently done through stool microscopy, most commonly Kato-Katz, with *A. lumbricoides* EPG categories of light (1–4999 EPG), moderate (5000–49,999 EPG) and heavy (>50,000 EPG) infections [3]. These thresholds need to be refined, and more research is required to determine the correlation between EPG calculated by FLOTAC, McMaster and Kato-Katz. Unlike some common NTDs, questionnaires are not a sensitive tool for identification of communities targeted for STH treatments [95].

#### Ideal Tools

Although current stool-based tests may be sufficient to define mass treatment strategies, especially in moderate- to high-endemic areas, tests with higher sensitivity are needed as infection intensity is reduced [4]. Similar to rapid, point-of-care diagnostic tests developed for other infectious diseases [48, 96, 97], mapping for STH control programmes need more convenient, reliable and affordable tools, including tests for detection of antigens, host immunological markers and/or parasite DNA, ideally in urine, blood or oral fluid [98–100]. However, due to the location of STHs in the intestines, it is possible that coproantigen tests will be more sensitive, although research to support this prediction is needed. Moreover, improved coordination of disease mapping, including specimens sampled for other NTD surveys, could strengthen cooperation between health and non-health sectors, as well as attract sustainable funding for control programmes [101, 102].

### Monitoring and Evaluating Impact of Anti-helminthic Treatment

#### Currently Available Tests

The impact of mass control programmes is currently evaluated through sentinel site surveys [3]. In some instances, evaluating impact through repeated mapping is conducted, although the value of comparing cross-sectional survey results, often with differing protocols and techniques, is debatable [101]. At present, stool-based microscopy, especially Kato-Katz, remains the main diagnostic test to evaluate impact of treatment, and outcomes include binary values of prevalence and cure rate (CR; recommended by WHO), and numeric values of EPG and egg reduction rates (ERRs). Although cost and ease of use have historically been more important than diagnostic sensitivity, especially for prevalence and CR, more sensitive tools may be needed as successful control programmes lead to reduced prevalence and intensity of infection.

#### Ideal Tools

As infection intensity decreases, measuring disease transmission becomes increasingly important, and direct markers of infection, including antigens, will be required. As integrated control programmes develop, increased precision of diagnostic tests may improve the interpretation of the effect of complementing interventions, such as WASH [101]. As albendazole is used to treat both LF and STH infections, disease impact surveys may be coordinated [103], and collection of the same, conveniently sampled specimens would improve data validity and cost-effectiveness. Alternatively, techniques including a preservation stage, such as FLOTAC®, could be incorporated, and stools processed at a central location [36]. However, LF surveillance will probably scale down as the disease becomes eliminated ahead of STH programmes, and rapid on-site tools for STH diagnosis are highly required.

Although the limitations of currently available STH stool tests may be overcome by adjusted reporting metrics [38••], a convenient point-of-care test is needed for M&E of STH control programmes, including ascariasis. Ideally, the test would also detect other common tropical diseases, such as malaria, through a multi-array platform [104, 105]. Although novel NTD diagnostic tools are currently moving towards urine and blood specimens [50, 102], coproantigen tests, such as those available for other intestinal infections [106–108], may be the most sensitive diagnostic marker in *A. lumbricoides* infection.

In areas where elimination of STH may become a target for control programmes, antigen, antibody and/or multiplex qPCR assays may improve detection of disease. However, unlike microscopy, PCR results do not correlate with morbidity, unless infection intensity is accounted for [71], and PCR remains prohibitively expensive at this stage.

### Measuring Drug Efficacy

#### Currently Available Tests

Drug resistance is not routinely monitored by STH control programmes. Although rarely detected to date, resistance to benzimidazoles may arise from parasite selection pressure due to high frequency of mono-drug treatment [109]. Few studies have assessed the accuracy of available coprological methods for estimating drug efficacy, either for CR or even more rarely for ERR, and tools for measuring drug efficacy are commonly neglected [110]. ERR determined by Kato-Katz is currently recommended for measuring anti-helminthic drug efficacy; however, other stool techniques may be more sensitive, including pooled stool samples which may reduce volume-to-weight ratio confounders, as well as the costs [21, 23, 26, 33, 111•, 112••, 113].

#### Ideal Tools

Despite high costs, the increased sensitivity and specificity of PCR and qPCR could help establish accurate baseline prevalence and determine measures of drug efficacy, although a better understanding of the correlation between EPG and worm burden with qPCR quantifications is required. We recommend that PCR methods [70••] are used in conjunction with Kato-Katz, and ideally an additional stool-based microscopy method, for accurate measurement of drug efficacy. Single-nucleotide polymorphisms, associated with drug resistance in veterinary nematodes, may be useful molecular markers to detect early resistance in human *A. lumbricoides* infections [109]; however, more studies are needed to clarify their phenotypic relevance. Finally, studies suggest that monitoring of drug resistance could be integrated with other NTD control programmes [102].

### Surveillance of Disease Elimination and Recrudescence

#### Currently Available Tests

In contrast to other NTDs, global targets for STH control programmes do not currently include elimination. Nevertheless, recent guidelines [49••] recommend the coordination of various NTD surveillance surveys, and reports have highlighted how the need for stool collection for STH diagnosis, as opposed to blood or urine, remains an important challenge [114]. In areas where elimination is relevant, antibody detection in young children may be an appropriate measure of active transmission, although identification of appropriate Ig isotypes is needed.

#### Ideal Tools

Serology and PCR-based diagnostic tests are currently being developed for other NTDs with *Ascaris* primers or antibodies as add-ons. In order to ensure integrated diagnosis for all STH species, it is essential that research on antigen, antibody and molecular-based diagnostic tools for *Ascaris* is not left behind.

## Conclusions

There is a paucity of data on novel, convenient diagnostics for ascariasis, even compared to other NTDs. Standardised protocols and validated diagnostics are required for assessing the epidemiological situation, burden of disease and drug efficacy. Based on an updated review of the literature, we have presented the currently available tools for clinical diagnosis and for field tests used in national control programmes. It is possible that *Ascaris* diagnostics will shift to more sensitive techniques, such as FLOTAC®, serological tests and qPCR, as areas of low-intensity infection become more common. As control programmes are scaled up, the shifting epidemiology of STH will need to be addressed, and quantitative rapid, point-of-care tests are required for successful control. Increased investments in research on *Ascaris* and other STHs is urgently needed for the development of simple and affordable diagnostic tools to support efforts to reduce human suffering caused by these infections.

## References

[1] Pullan RL, Smith JL, Jasrasaria R, Brooker SJ (2014). Global numbers of infection and disease burden of soil transmitted helminth infections in 2010. Parasites Vectors.

[2] Taylor-Robinson DC, Maayan N, Soares-Weiser K, Donegan S, Garner P (2015). Deworming drugs for soil-transmitted intestinal worms in children: effects on nutritional indicators, haemoglobin, and school performance. Cochrane Database of Syst Rev.

[3] World Health Organization (2006) Preventative chemotherapy in human helminthiasis: coordinated use of anthelminthic drugs in control interventions: a manual for health professionals and programme managers. Available: [http://whqlibdoc.who.int/publications/2006/9241547103_eng.pdf], accessed: 08 July 2015.

[4] World Health Organization (2015) Investing to overcome the global impact of neglected tropical diseases. Third WHO report on neglected tropical diseases. Available: [http://www.who.int/neglected_diseases/9789241564861/en/], accessed: 03 August 2015.

[5] Brooker SJ, Bundy DAP (2014) Soil-transmitted Helminths (Geohelminths). In: Farrar J, Hotez PJ, Junghanss T, Kang G, Lalloo DG et al., editors. Manson’s Tropical Diseases. 23^rd^ ed: Elsevier Saunders. pp. 766-776.

[6] Nejsum P, Parker ED, Frydenberg J, Roepstorff A, Boes J (2005). Ascariasis is a zoonosis in Denmark. J Clin Microbiol.

[7] Lejkina ES (1965). Research on ascariasis immunity and immunodiagnosis. Bull World Health Organ.

[8] Şentürk T, Özdemir B, Keçebaş M, Beşli F, Yesilbursa D (2012). *Ascaris*-induced eosinophilic myocarditis presenting as acute ST elevation myocardial infarction and cardiogenic shock in a young woman. J Cardiovasc Med.

[9] Baba AA, Ahmad SM, Sheikh KA (2009). Intestinal ascariasis: the commonest cause of bowel obstruction in children at a tertiary care center in Kashmir. Pediatr Surg Int.

[10] Das AK (2014). Hepatic and biliary ascariasis. J Global Infect Dis.

[11] Tortajada-Laureiro L, Olveira-Martin A, Marin-Serrano E, Ruiz-Fernandez G, Eun JH (2012). Biliary parasite (*Ascaris*) as a cause of acute pancreatitis. Ultrasound Diagn Rev Esp Enferm Dig.

[12] Aydin R, Bekci T, Bilgici MC, Polat AV (2014). Sonographic diagnosis of ascariasis causing small bowel obstruction. J Clin Ultrasound.

[13] Roy S, Karmacharya P, Aryal MR (2014) Diagnosis of intestinal ascariasis in modern era. BMJ Case Reports 2014. 10.1136/bcr-2013-200529 PMC3992599 24739652

[14] Yamashita ET, Takahashi W, Kuwashima DY, Langoni TR, Costa-Genzini A (2013). Diagnosis of *Ascaris lumbricoides* infection using capsule endoscopy. World J Gastrointest Endosc.

[15] Wickramasinghe DP, Samarasekera DN (2012). Intestinal helminths detected in capsule endoscopy. Dig Endosc.

[16] Papier K, Williams GM, Luceres-Catubig R, Ahmed F, Olveda RM (2014). Childhood malnutrition and parasitic helminth interactions. Clin Infect Dis.

[17] Hagel I, Lynch NR, Puccio F, Rodriguez O, Luzondo R (2003). Defective regulation of the protective IgE response against intestinal helminth *Ascaris lumbricoides* in malnourished children. J Trop Pediatr.

[18] Katz N, Chaves A, Pellegrino J (1972). A simple device for quantitative stool thick-smear technique in schistosomiasis mansoni. Rev Inst Med Trop Sao Paulo.

[19] Sinniah B (1982). Daily egg production of *Ascaris lumbricoides*: the distribution of eggs in the faeces and the variability of egg counts. Parasitology.

[20.•] Speich B, Ali SM, Ame SM, Albonico M, Utzinger J (2015). Quality control in the diagnosis of *Trichuris trichiura* and *Ascaris lumbricoides* using the Kato-Katz technique: experience from three randomised controlled trials. Parasites Vectors.

[21] Albonico M, Rinaldi L, Sciascia S, Morgoglione ME, Piemonte M (2013). Comparison of three copromicroscopic methods to assess albendazole efficacy against soil-transmitted helminth infections in school-aged children on Pemba Island. Trans R Soc Trop Med Hyg.

[22.•] Nikolay B, Brooker SJ, Pullan RL (2014). Sensitivity of diagnostic tests for human soil-transmitted helminth infections: a meta-analysis in the absence of a true gold standard. Int J Parasitol.

[23] Levecke B, Behnke JM, Ajjampur SS, Albonico M, Ame SM (2011). A comparison of the sensitivity and fecal egg counts of the McMaster egg counting and Kato-Katz thick smear methods for soil-transmitted helminths. PLoS Negl Trop Dis.

[24] Knopp S, Mgeni AF, Khamis IS, Steinmann P, Stothard JR (2008). Diagnosis of soil-transmitted helminths in the era of preventive chemotherapy: effect of multiple stool sampling and use of different diagnostic techniques. PLoS Negl Trop Dis.

[25] Coulibaly JT, Furst T, Silue KD, Knopp S, Hauri D (2012). Intestinal parasitic infections in schoolchildren in different settings of Côte d’Ivoire: effect of diagnostic approach and implications for control. Parasites Vectors.

[26] Speich B, Utzinger J, Marti H, Ame SM, Ali SM (2014). Comparison of the Kato-Katz method and ether-concentration technique for the diagnosis of soil-transmitted helminth infections in the framework of a randomised controlled trial. Eur J Clin Microbiol Infect Dis.

[27] Sayasone S, Utzinger J, Akkhavong K, Odermatt P (2015). Repeated stool sampling and use of multiple techniques enhance the sensitivity of helminth diagnosis: a cross-sectional survey in southern Lao People’s Democratic Republic. Acta Trop.

[28] Habtamu K, Degarege A, Ye-Ebiyo Y, Erko B (2011). Comparison of the Kato-Katz and FLOTAC techniques for the diagnosis of soil-transmitted helminth infections. Parasitol Int.

[29] Knopp S, Rinaldi L, Khamis IS, Stothard JR, Rollinson D (2009). A single FLOTAC is more sensitive than triplicate Kato-Katz for the diagnosis of low-intensity soil-transmitted helminth infections. Trans R Soc Trop Med Hyg.

[30] Knopp S, Speich B, Hattendorf J, Rinaldi L, Mohammed KA (2011). Diagnostic accuracy of Kato-Katz and FLOTAC for assessing anthelmintic drug efficacy. PLoS Negl Trop Dis.

[31] Glinz D, Silue KD, Knopp S, Lohourignon LK, Yao KP (2010). Comparing diagnostic accuracy of Kato-Katz, Koga agar plate, ether-concentration, and FLOTAC for *Schistosoma mansoni* and soil-transmitted helminths. PLoS Negl Trop Dis.

[32] Barda BD, Rinaldi L, Ianniello D, Zepherine H, Salvo F (2013). Mini-FLOTAC, an innovative direct diagnostic technique for intestinal parasitic infections: experience from the field. PLoS Negl Trop Dis.

[33] Barda B, Cajal P, Villagran E, Cimino R, Juarez M (2014). Mini-FLOTAC, Kato-Katz and McMaster: three methods, one goal; highlights from north Argentina. Parasites Vectors.

[34] Assefa LM, Crellen T, Kepha S, Kihara JH, Njenga SM (2014). Diagnostic accuracy and cost-effectiveness of alternative methods for detection of soil-transmitted helminths in a post-treatment setting in western Kenya. PLoS Negl Trop Dis.

[35] Jeandron A, Abdyldaieva G, Usubalieva J, Ensink JH, Cox J (2010). Accuracy of the Kato-Katz, adhesive tape and FLOTAC techniques for helminth diagnosis among children in Kyrgyzstan. Acta Trop.

[36] Barda B, Albonico M, Ianniello D, Ame SM, Keiser J (2015). How long can stool samples be fixed for an accurate diagnosis of soil-transmitted helminth infection using Mini-FLOTAC?. PLoS Negl Trop Dis.

[37] Speich B, Knopp S, Mohammed KA, Khamis IS, Rinaldi L (2010). Comparative cost assessment of the Kato-Katz and FLOTAC techniques for soil-transmitted helminth diagnosis in epidemiological surveys. Parasites Vectors.

[38.••] Levecke B, Brooker SJ, Knopp S, Steinmann P, Sousa-Figueiredo JC (2014). Effect of sampling and diagnostic effort on the assessment of schistosomiasis and soil-transmitted helminthiasis and drug efficacy: a meta-analysis of six drug efficacy trials and one epidemiological survey. Parasitology.

[39] Carvalho GL, Moreira LE, Pena JL, Marinho CC, Bahia MT (2012). A comparative study of the TF-Test®, Kato-Katz, Hoffman-Pons-Janer, Willis and Baermann-Moraes coprologic methods for the detection of human parasitosis. Mem Inst Oswaldo Cruz.

[40] Gonçalves AQ, Abellana R, Pereira-da-Silva HD, Santos I, Serra PT (2014). Comparison of the performance of two spontaneous sedimentation techniques for the diagnosis of human intestinal parasites in the absence of a gold standard. Acta Trop.

[41] Funk AL, Boisson S, Clasen T, Ensink JH (2013). Comparison of Kato-Katz, ethyl-acetate sedimentation, and Midi Parasep® in the diagnosis of hookworm, *Ascaris* and *Trichuris* infections in the context of an evaluation of rural sanitation in India. Acta Trop.

[42] Alfredo Fernández-Nino J, David Ramírez J, Consuelo López M, Inés Moncada L, Reyes P (2015). Agreement of the Kato-Katz test established by the WHO with samples fixed with sodium acetate analyzed at 6 months to diagnose intestinal geohelminthes. Acta Trop.

[43] Machicado JD, Marcos LA, Tello R, Canales M, Terashima A (2012). Diagnosis of soil-transmitted helminthiasis in an Amazonic community of Peru using multiple diagnostic techniques. Trans R Soc Trop Med Hyg.

[44] Goodman D, Haji HJ, Bickle QD, Stoltzfus RJ, Tielsch JM, et al. A comparison of methods for detecting the eggs of *Ascaris*, *Trichuris*, and Hookworm in infant stool, and the epidemiology of infection in Zanzibari infants. Am J Trop Med Hyg. 2007;76:725–31. 17426179

[45] Bogoch II, Andrews JR, Speich B, Utzinger J, Ame SM, et al. Mobile phone microscopy for the diagnosis of soil-transmitted helminth infections: a proof-of-concept study. Am J Trop Med Hyg. 2013;88:626–9. 10.4269/ajtmh.2013.12-0742 PMC3617844 23478580

[46.•] Bogoch II, Coulibaly JT, Andrews JR, Speich B, Keiser J, et al. Evaluation of portable microscopic devices for the diagnosis of *Schistosoma* and soil-transmitted helminth infection. Parasitology. 2014;141:1811–8. **Evaluation of novel, field-friendly devices found sufficient diagnostic accuracy for** *T. trichiura***and** *S. mansoni***, but not for** *A. lumbricoides*. 10.1017/S0031182014000432 24776232

[47] Tuijn CJ, Hoefman BJ, van Beijma H, Oskam L, Chevrollier N (2011). Data and image transfer using mobile phones to strengthen microscopy-based diagnostic services in low and middle income country laboratories. PLoS One.

[48] Mudanyali O, Dimitrov S, Sikora U, Padmanabhan S, Navruz I (2012). Integrated rapid-diagnostic-test reader platform on a cellphone. Lab Chip.

[49.••] World Health Organization (2015) Assessing the epidemiology of soil-transmitted helminths during a transmission assessment survey in the Global programme for the elimination of the lymphatic filariasis. Available at [http://apps.who.int/iris/bitstream/10665/153240/1/9789241508384_eng.pdf], accessed: 04 August 2015. **Recent WHO guidelines for integration of neglected tropical disease (NTD) control programme surveys for lymphatic filariasis and soil-transmitted helminth infections.**

[50] Colley DG, Binder S, Campbell C, King CH, Tchuem Tchuenté LA (2013). A five-country evaluation of a point-of-care circulating cathodic antigen urine assay for the prevalence of *Schistosoma mansoni*. Am J Trop Med Hyg.

[51] Haswell-Elkins MR, Leonard H, Kennedy MW, Elkins DB, Maizels RM (1992). Immunoepidemiology of *Ascaris lumbricoides*: relationships between antibody specificities, exposure and infection in a human community. Parasitology.

[52] King EM, Kim HT, Dang NT, Michael E, Drake L (2005). Immuno-epidemiology of *Ascaris lumbricoides i*nfection in a high transmission community: antibody responses and their impact on current and future infection intensity. Parasite Immunol.

[53] Hagel I, Cabrera M, Buvat E, Gutierrez L, Santaella C (2008). Antibody responses and resistance against *Ascaris lumbricoides* infection among Venezuelan rural children: the influence of ethnicity. J Trop Pediatr.

[54] Cooper PJ, Chico ME, Sandoval C, Nutman TB (2004). Atopic phenotype is an important determinant of immunoglobulin E-mediated inflammation and expression of T helper cell type 2 cytokines to *Ascaris* antigens in children exposed to ascariasis. J Infect Dis.

[55] Chatterjee BP, Santra A, Karmakar PR, Mazumder DN (1996). Evaluation of IgG4 response in ascariasis by ELISA for serodiagnosis. Tropical Med Int Health.

[56] Bhattacharyya T, Santra A, Majumder DN, Chatterjee BP (2001). Possible approach for serodiagnosis of ascariasis by evaluation of immunoglobulin G4 response using *Ascaris lumbricoides* somatic antigen. J Clin Microbiol.

[57] Hagan P, Blumenthal UJ, Dunn D, Simpson AJ, Wilkins HA (1991). Human IgE, IgG4 and resistance to reinfection with *Schistosoma haematobium*. Nature.

[58] Burk SV, Dangoudoubiyam S, Brewster-Barnes T, Bryant UK, Howe DK (2014). *In vitro* culture of *Parascaris equorum* larvae and initial investigation of parasite excretory-secretory products. Parasitol Res.

[59] Acevedo N, Mohr J, Zakzuk J, Samonig M, Briza P (2013). Proteomic and immunochemical characterization of glutathione transferase as a new allergen of the nematode *Ascaris lumbricoides*. PLoS One.

[60] Santra A, Bhattacharya T, Chowdhury A, Ghosh A, Ghosh N (2001). Serodiagnosis of ascariasis with specific IgG4 antibody and its use in an epidemiological study. Trans R Soc Trop Med Hyg.

[61] Needham CS, Lillywhite JE, Beasley NM, Didier JM, Kihamia CM (1996). Potential for diagnosis of intestinal nematode infections through antibody detection in saliva. Trans R Soc Trop Med Hyg.

[62] Lamberton PH, Kabatereine NB, Oguttu DW, Fenwick A, Webster JP (2014). Sensitivity and specificity of multiple Kato-Katz thick smears and a circulating cathodic antigen test for *Schistosoma mansoni* diagnosis pre- and post-repeated-praziquantel treatment. PLoS Negl Trop Dis.

[63] Moss DM, Priest JW, Hamlin K, Derado G, Herbein J (2014). Longitudinal evaluation of enteric protozoa in Haitian children by stool exam and multiplex serologic assay. Am J Trop Med Hyg.

[64] Zakzuk J, Acevedo N, Cifuentes L, Bornacelly A, Sanchez J (2013). Early life IgE responses in children living in the tropics: a prospective analysis. Pediatr Allergy Immunol.

[65] Hall A, Romanova T (1990). *Ascaris lumbricoides*: detecting its metabolites in the urine of infected people using gas-liquid chromatography. Exp Parasitol.

[66] Carlsgart J, Roepstorff A, Nejsum P (2009). Multiplex PCR on single unembryonated *Ascaris* (roundworm) eggs. Parasitol Res.

[67] Jaeger LH, Iñiguez AM (2014). Molecular paleoparasitological hybridization approach as effective tool for diagnosing human intestinal parasites from scarce archaeological remains. PLoS One.

[68] Phuphisut O, Yoonuan T, Sanguankiat S, Chaisiri K, Maipanich W (2014). Triplex polymerase chain reaction assay for detection of major soil-transmitted helminths, *Ascaris lumbricoides*, *Trichuris trichiura*, *Necator americanus*, in fecal samples. Southeast Asian J Trop Med Public Health.

[69] Basuni M, Muhi J, Othman N, Verweij JJ, Ahmad M (2011). A pentaplex real-time polymerase chain reaction assay for detection of four species of soil-transmitted helminths. Am J Trop Med Hyg.

[70.••] Mejia R, Vicuña Y, Broncano N, Sandoval C, Vaca M, et al. A novel, multi-parallel, real-time polymerase chain reaction approach for eight gastrointestinal parasites provides improved diagnostic capabilities to resource-limited at-risk populations. Am J Trop Med Hyg. 2013;88:1041–7. **A novel multiplex real-time polymerase chain reaction (PCR) assay performed better than direct microscopy for*****Ascaris*****and*****Giardia*****in stool samples from children before and after anti-helminthic treatment.** 10.4269/ajtmh.12-0726 PMC3752800 23509117

[71] Arndt MB, John-Stewart G, Richardson BA, Singa B, van Lieshout L (2013). Impact of helminth diagnostic test performance on estimation of risk factors and outcomes in HIV-positive adults. PLoS One.

[72] Gordon CA, McManus DP, Acosta LP, Olveda RM, Williams GM (2015). Multiplex real-time PCR monitoring of intestinal helminths in humans reveals widespread polyparasitism in Northern Samar, the Philippines. Int J Parasitol.

[73] Taniuchi M, Verweij JJ, Noor Z, Sobuz SU, Lieshout L, et al. High throughput multiplex PCR and probe-based detection with Luminex beads for seven intestinal parasites. Am J Trop Med Hyg. 2011;84:332–7. 10.4269/ajtmh.2011.10-0461 PMC3029193 21292910

[74] Fitzpatrick JM, Peak E, Perally S, Chalmers IW, Barrett J (2009). Anti-schistosomal intervention targets identified by lifecycle transcriptomic analyses. PLoS Negl Trop Dis.

[75] Quilès F, Balandier JY, Capizzi-Banas S. In situ characterisation of a microorganism surface by Raman microspectroscopy: the shell of *Ascaris* eggs. Anal Bioanal Chem. 2006;386:249–55. 10.1007/s00216-006-0638-4 16900382

[76] Kweon OJ, Choi JH, Song UH, Park AJ (2015). Performance evaluation of a DNA chip assay in the identification of major genitourinary pathogens. J Microbiol Methods.

[77] Criscione CD, Anderson JD, Sudimack D, Peng W, Jha B (2007). Disentangling hybridization and host colonization in parasitic roundworms of humans and pigs. Proc Royal Soc Biol Sci.

[78] Criscione C (2013) Genetic epidemiology of *Ascaris*: Cross-transmission between humans and pigs, focal transmission, and effective population size. In: Holland C, editor. *Ascaris*: the neglected parasite. Available at [http://www.bio.tamu.edu/USERS/criscione/publications/13_criscione%20Ascaris%20BC%202013.pdf], accessed 26 November 2014: Elsevier. pp. 203-231.

[79] Betson M, Nejsum P, Bendall RP, Deb RM, Stothard JR (2014) Molecular epidemiology of ascariasis: a global perspective on the transmission dynamics of *Ascaris* in people and pigs. J Infect Dis. 10.1093/infdis/jiu193 PMC4136802 24688073

[80] Nejsum P, Betson M, Bendall RP, Thamsborg SM, Stothard JR (2012). Assessing the zoonotic potential of *Ascaris suum* and *Trichuris suis*: looking to the future from an analysis of the past. J Helminthol.

[81] Pinelli E, Willers SM, Hoek D, Smit HA, Kortbeek LM (2009). Prevalence of antibodies against *Ascaris suum* and its association with allergic manifestations in 4-year-old children in The Netherlands: the PIAMA birth cohort study. Eur J Clin Microbiol Infect Dis.

[82] Zhou C, Yuan K, Tang X, Hu N, Peng W (2011). Molecular genetic evidence for polyandry in *Ascaris suum*. Parasitol Res.

[83] Larsen M (1999). Biological control of helminths. Int J Parasitol.

[84] Ansel M, Thibaut M (1973). Value of the specific distinction between *Ascaris lumbricoides* Linnaeus 1758 and *Ascaris suum* Goeze 1782. Int J Parasitol.

[85] Schneider R, Obwaller A, Auer H (2015). Immunoblot for the detection of *Ascaris suum*-specific antibodies in patients with visceral larva migrans (VLM) syndrome. Parasitol Res.

[86] Vlaminck J, Nejsum P, Vangroenweghe F, Thamsborg SM, Vercruysse J (2012). Evaluation of a serodiagnostic test using *Ascaris suum* haemoglobin for the detection of roundworm infections in pig populations. Vet Parasitol.

[87.•] Vlaminck J, Levecke B, Vercruysse J, Geldhof P. Advances in the diagnosis of *Ascaris suum* infections in pigs and their possible applications in humans. Parasitology. 2014;141:1904–11. **Review of current diagnostic methods for detection of*****A. suum*****infections in humans and pigs, including novel serological techniques.** 10.1017/S0031182014000328 24775944

[88] Leles D, Araújo A, Vicente AC, Iñiguez AM (2010). ITS1 intra-individual variability of *Ascaris* isolates from Brazil. Parasitol Int.

[89] Liu GH, Wu CY, Song HQ, Wei SJ, Xu MJ (2012). Comparative analyses of the complete mitochondrial genomes of *Ascaris lumbricoides* and *Ascaris suum* from humans and pigs. Gene.

[90] Park YC, Kim W, Park JK (2011). The complete mitochondrial genome of human parasitic roundworm, *Ascaris lumbricoides*. Mitochondrial DNA.

[91] Okimoto R, Macfarlane JL, Clary DO, Wolstenholme DR (1992). The mitochondrial genomes of two nematodes, *Caenorhabditis elegans* and *Ascaris suum*. Genetics.

[92] Jex AR, Liu S, Li B, Young ND, Hall RS (2011). *Ascaris suum* draft genome. Nature.

[93] Leles D, Gardner SL, Reinhard K, Iñiguez A, Araújo A (2012). Are *Ascaris lumbricoides* and *Ascaris suum* a single species?. Parasites Vectors.

[94] Peng W, Criscione CD (2012). Ascariasis in people and pigs: new inferences from DNA analysis of worm populations. Infect Genet Evol.

[95] Booth M, Mayombana C, Machibya H, Masanja H, Odermatt P (1998). The use of morbidity questionnaires to identify communities with high prevalences of schistosome or geohelminth infections in Tanzania. Trans R Soc Trop Med Hyg.

[96] Bradley JE, Gillespie AJ, Trenholme KR, Karam M (1993). The effects of vector control on the antibody response to antigens of *Onchocerca volvulus*. Parasitology.

[97] Ramachandran CP (1993). Improved immunodiagnostic tests to monitor onchocerciasis control programmes - a multicenter effort. Parasitol Today.

[98] Shonhai A, Warrener L, Mangwanya D, Slibinskas R, Brown K, et al. Investigation of a measles outbreak in Zimbabwe, 2010: potential of a point of care test to replace laboratory confirmation of suspected cases. Epidemiol Infect. 2015;1–9. 10.1017/S0950268815000540 PMC9150933 25865645

[99] Tebruegge M, Dutta B, Donath S, Ritz N, Forbes B, et al. (2015) Mycobacteria-specific cytokine responses detect TB infection and distinguish latent from active TB. Am J Respir Crit Care Med. 10.1164/rccm.201501-0059OC 26030187

[100] Rayment M, Doku E, Thornton A, Pearn M, Sudhanva M (2013). Automatic oral fluid-based HIV testing in HIV screening programmes: automatic for the people. HIV Medicine.

[101] King JD, Endeshaw T, Escher E, Alemtaye G, Melaku S (2013). Intestinal parasite prevalence in an area of Ethiopia after implementing the SAFE strategy, enhanced outreach services, and health extension program. PLoS Negl Trop Dis.

[102] Pion SD, Chesnais CB, Bopda J, Louya F, Fischer PU (2015). The impact of two semiannual treatments with albendazole alone on lymphatic filariasis and soil-transmitted helminth infections: a community-based study in the Republic of Congo. AmJTrop Med Hyg.

[103] Smith JL, Sturrock HJ, Assefa L, Nikolay B, Njenga SM (2015). Factors associated with the performance and cost-effectiveness of using lymphatic filariasis transmission assessment surveys for monitoring soil-transmitted helminths: a case study in Kenya. Am J Trop Med Hyg.

[104] Salim N, Knopp S, Lweno O, Abdul U, Mohamed A (2015). Distribution and risk factors for *Plasmodium* and helminth co-infections: a cross-sectional survey among children in Bagamoyo district, coastal region of Tanzania. PLoS Negl Trop Dis.

[105] Mwandawiro CS, Nikolay B, Kihara JH, Ozier O, Mukoko DA (2013). Monitoring and evaluating the impact of national school-based deworming in Kenya: study design and baseline results. Parasites Vectors.

[106] Llorente MT, Clavel A, Varea M, Olivera S, Castillo FJ (2002). Evaluation of an immunochromatographic dip-strip test for the detection of *Cryptosporidium* oocysts in stool specimens. Eur J Clin Microbiol Infect Dis.

[107] Pillai DR, Kain KC (1999). Immunochromatographic strip-based detection of *Entamoeba histolytica*-*E. dispar* and *Giardia lamblia* coproantigen. J Clin Microbiol.

[108] Sykes AM, McCarthy JS (2011). A coproantigen diagnostic test for *Strongyloides* infection. PLoS Negl Trop Dis.

[109] Diawara A, Schwenkenbecher JM, Kaplan RM, Prichard RK (2013). Molecular and biological diagnostic tests for monitoring benzimidazole resistance in human soil-transmitted helminths. Am J Trop Med Hyg.

[110] Lammie PJ, Solomon AW, Secor WE, Peeling R (2011) A14 Diagnostic needs for NTD programs. The causes and impacts of neglected tropical and zoonotic diseases Opportunities for integrated intervention strategies Institute of Medicine (US) Forum on Microbial Threats: Washington (DC): National Academies Press (US). 21977543

[111.•] Mekonnen Z, Meka S, Ayana M, Bogers J, Vercruysse J, et al. Comparison of individual and pooled stool samples for the assessment of Soil-Transmitted helminth infection intensity and drug efficacy. PLoS Negl Trop Dis. 2013;7, e2189. **Comparative study of faecal egg count (FEC) by McMaster egg counting method in individual and pooled stool samples found a significant positive correlation between results, apart from 60 samples for*****A. lumbricoides.*** 10.1371/journal.pntd.0002189 PMC3656117 23696905

[112.••] McCarthy JS, Lustigman S, Yang GJ, Barakat RM, Garcia HH (2012). A research agenda for helminth diseases of humans: diagnostics for control and elimination programmes. PLoS Negl Trop Dis.

[113] Speich B, Ali SM, Ame SM, Bogoch II, Alles R (2015). Efficacy and safety of albendazole plus ivermectin, albendazole plus mebendazole, albendazole plus oxantel pamoate, and mebendazole alone against *Trichuris trichiura* and concomitant soil-transmitted helminth infections: a four-arm, randomised controlled trial. Lancet Infect Dis.

[114] Gunawardena S, Gunawardena NK, Kahathuduwa G, Karunaweera ND, de Silva NR (2014). Integrated school-based surveillance for soil-transmitted helminth infections and lymphatic filariasis in Gampaha district, Sri Lanka. Am J Trop Med Hyg.

